# Mr. Crowfoot's Case of Apoplexy

**Published:** 1801-12

**Authors:** W. Crowfoot

**Affiliations:** Beccles


### *Mr. Crowfoot's Case of Apoplexy.*

ON the firft perufal of the following Statement, and the Anfwer to it, we thought it bore too much of the character of a *personal* difpute to be admitted. Confidering, however, that many of our Readers may be divided in their opinions on this important difeafe, and that a knowledge of the true caufes of Apoplexy, and the moft rational mode of treating it, cannot be too foon or too widely diffufed, we have fubmitted it to our Readers in its prefent form, in order to collect their fentiments on the fubject. For this reafon, we forbear giving any opinion of our own at prefent.

A STATEMENT

### *A STATEMENT of a certain Case of APOPLEXY*, Signed WILLIAM CROWFOOT.

ON Wednefday the 26th of Auguft, 1801, a lady, aged 59, was feized, almoft as foon as dinner was ended, with an attack of Apoplexy.*

Mr. P. who had been called in, had blooded the patient, and applied blifters to the arms. It was between feven and eight o'clock when I arrived, and found the lady much better, but complaining of head-ach and ficknefs. I agreed with Mr. P. in the propriety of emptying the ftomach by an emetic.†

But Dr. L. being expected every inftant, and the patient very much relieved, I thought it unneceffary to do any thing farther before he came. I now went down ftairs, and requefted to take my leave; the lady being materially recovered, I did not conceive my prefence effential to her well-doing. I had fubftantial reafons for avoiding an interview with the Doctor, (and reafons I am very willing to declare both to himfelf and others) but the gentleman of the houfe being defirous I fhould not leave B. gave me an offer (knowing thefe objections) that I fhould retire to another room. I acquiefced; but before the neceffary arrangement could take place, the very man I had been fo ftudious to efcape from, prefented himfelf at the door; we were, in truth, taken by furprife; and the gentleman, unwilling to wound my feelings by an introduction, fuppreffed my name as being prefent, and thereby placed me in a very awkward and uncomfortable fituation. After a few minutes abfence, and a firft fight of the patient, the Doctor returned with a decided opinion as to the nature of the cafe, and afferted, in the moft pofitive language, that there was a confiderable extravafation of blood upon the brain, and that the ficknefs was the effect of compreffion, &c. He declared, that an emetic, which he underftood was propofed to be given, was a moft injudicious remedy; that vomiting caufed a very great determination to the head, and the action of vomiting would but increafe the complaint; the ficknefs in this cafe was merely fymptomatic, the fame as in violent injuries of the head, wherein vomiting always fupervened, &c. It may be fuppofed, that

* This is called Apoplexy, to avoid all occafion of cavilling. I was not prefent during the paroxyfm, but I underftood the patient was much convulfed.

† With regard to the propriety of emetics in cafes of Apoplexy, I beg to refer to an excellent paper on that fubject by Dr. Fothergill. Vide Medical Obfervations and Inquiries, vol. v. p. 80.

during this explanation of the patient's fituation, my imagined concealment was extremely irkfome to me, and muft have been borne with great impatience; for I was thus made a witnefs to converfation in which my conduct was concerned, without the power of making any reply.*

It became infupportable, and I announced myfelf with affuring the Doctor, I was incapable of performing fo vile an action as that of a fpy, and how accidentally, as well as unintentionally on my part, I had thus fallen into his company. The gentleman declared he had not mentioned my being there, becaufe he thought I fhould diflike it. I faid, I certainly had objections to meeting Dr. L. for reafons which the time and place did not allow me to explain; but having met, I could not fubmit to any imputation on my character, which my filence might occafion; indeed, it appeared a dereliction of my duty, which now called for a facrifice of my private feeling, and I thought it would be highly difhonourable to fhrink from fuch a difclofure. The Doctor, upon this, expreffed his defire to be upon good terms with every medical man; and to prove his fincerity, affured me, he would go fifty miles to ferve poor Revans; adverting then to the patient. He fpoke of applying leeches; and turning towards me, anfwered for my concurrence in the propriety of his advice. I told him I did think topical bleeding very proper, and for the reafon he had juft affigned; that is to fay, that it would not weaken like general bleeding: But I did not agree with him, by any means, in his doctrine of extravafation; for, in my judgment, a confiderable extravafation, and confequently, confiderable compreffion, muft produce fymptoms of the moft formidable nature; and which fymptoms, in the cafe above ftairs, did not appear to exift. The patient at this moment was fenfible and collected; fhe remembered not only writing a note to me the day before, but alfo the occafion of writing, which fhe explained to me; there was no mark of paralyfis, or interruption of the vital functions; no appearance of oppreffion in the countenance or the pulfe: And in the hope to quiet the alarm occafioned by the Doctor's declaration to the gentleman, I took the liberty to affure him, upon taking leave, there was no extravafation, and my confident hope that we fhould find her better in the morning.

Thurfday, Auguft 27. Our patient was better; fhe paffed a very good night; the leeches had not been applied, thofe procured

* Mr. P. was at this time in the fick chamber above ftairs, and of courfe could have no opportunity of defending himfelf.

cured being thought but indifferent, and becaufe the patient was manifeftly much amended; they were, however, ufed this morning.

Friday, Auguft 28. Continued in the ftate of convalefcence. I thought the idea of extravafation muft be relinquifhed; and in converfation this morning, in prefence of the gentleman and Mr. P. the fubject was brought again under difcuffion. The Doctor was of opinion very little remained, but he expreffed his conviction of it in the firft inftance; and in every cafe of Apoplexy, he never knew, he faid, any cafe where there was an abolition of the fenfes, the jaw became locked, and the hand fell down, but it was occafioned by extravafation. I afked him if he had ever feen a cafe of Epilepfy? He faid, he hoped I diftinguifhed between Apoplexy and Epilepfy. To which I replied, I only meant to prove his affertion unfounded, that the abolition of the fenfes was always the confequence of extravafation. I told him I was perfectly aftonifhed to hear him declare fo pofitively, after firft feeing the patient, that there was confiderable extravafation of blood upon the brain. He anfwered, he believed he did not fay confiderable, and that it might rather be exudation than extravafation. I told the Doctor, he might call it which he pleafed; if it was confiderable, compreffion muft be the confequence, and in my opinion it was a diftinction without a difference. But I affured him he ufed the expreffion of *considerable extravasation*, and that the opinion was directly in oppofition to my judgment. Dr. L. acknowledged the beft Anatomifts were of different opinions on the fubject, but that he had diffected many, and had always obferved it to arife from extravafation. The gentleman here clofed the converfation by obferving, that all thofe Dr. L. had diffected were fatal cafes, and he hoped the head in queftion would not require fuch an examination.

W. CROWFOOT.

*Beccles, Sept.* 12, 1801.

There was a Note, which Dr. L. had not time to copy, containing an obfervation of Mr. Crowfoot, that if extravafation had ever really taken place in the cafe of this lady, that fhe certainly would not have been in a fituation, at the time we met, to require any affiftance from us; meaning, as Mr. P. and myfelf fuppofed, that in *all cases of extravasation on the brain death ensues.* If our fuppofition is wrong, Mr. C. will have the goodnefs to explain himfelf.

---

The

The following is a Copy of Dr. FOTHERGILL's Paper upon Apoplexy, alluded to by Mr. CROWFOOT in his Statement.

IT is inculcated by Dr. Fothergill, in the paper before referred to, that the obvious caufe of Apoplexy, in the greater number of cafes, arifes from the ftomach, and therefore the affection in the head is fymptomatic; and in conformity with this doctrine, he fays, We are as fpeedily as poffible to endeavour, by all the means we can, to remove the load by emetics, purgatives, &c.

We need not, fays he, be under much reftraint in the ufe of thefe remedies, till thorough evacuations are procured; the ftimulus excited in the ftomach, and the room provided by a freer circulation, are almoft alike beneficial, and without diminifhing the patient's ftrength, make way for his recovery. I am of opinion, fays the fame gentleman, that we fhould probably render the moft effectual aid by endeavouring, in all cafes, to procure a plentiful difcharge from the ftomach and bowels, as by thefe revulfions the head is never more relieved from plenitude, and that without weakening or interruption of other efforts of Nature to relieve herfelf.

---

There was alfo another Note, containing the cafe of a Mrs. N. who was attacked with Apoplexy, and attended by Mr. D. who cured her by judicioufly adminiftering emetics, as ftated in this note by Mr. C. Vide Dr. L's comment on this note.

